# Can isometric testing substitute for the one repetition maximum squat test?

**DOI:** 10.1007/s00421-024-05554-8

**Published:** 2024-08-05

**Authors:** Konstantin Warneke, Michael Keiner, David G. Behm, Klaus Wirth, Martin Kaufmann, Mareike Sproll, Andreas Konrad, Sebastian Wallot, Martin Hillebrecht

**Affiliations:** 1 Institute of Human Movement Science, Sport and Health, University of Graz, Graz, Austria; 2 Institute of Exercise Science, German University of Sport and Health, Ismaning, Germany; 3 School of Human Kinetics and Recreation, Memorial University of Newfoundland, St. John’s, NL Canada; 4 Institute of Exercise Science, University of Applied Sciences Wiener Neustadt, Wiener Neustadt, Austria; 5 University Sports Center, Carl von Ossietzky University Oldenburg, Oldenburg, Germany; 6 Department of Human Motion Science and Exercise Physiology, Friedrich Schuller University, Jena, Germany; 7 Institute of Psychology, Leuphana University Lüneburg, Lüneburg, Germany

**Keywords:** Maximal strength, Squat, Isometric mid-thigh pull, Agreement, Concordance, Testing specificity

## Abstract

When measuring maximum strength, a high accuracy and precision is required to monitor the training adaptations. Based on available reliability parameters, the literature suggests the replacement of the one repetition maximum (1RM) by isometric testing to save testing time. However, from a statistical point of view, correlation coefficients do not provide the required information when aiming to replace one test by another. Therefore, the literature suggests the inclusion of the mean absolute error (MAE), the mean absolute percentage error (MAPE) for agreement analysis. Consequently, to check the replaceability of 1RM testing methods, the current study examined the agreement of isometric and dynamic testing methods in the squat and the isometric mid-thigh pull. While in accordance with the literature, correlations were classified high *r* = 0.638–0.828 and ICC = 0.630–0.828, the agreement analysis provided MAEs of 175.75–444.17 N and MAPEs of 16.16–57.71% indicating an intolerable high measurement error between isometric and dynamic testing conditions in the squat and isometric mid-thigh pull. In contrast to previous studies, using MAE, MAPE supplemented by CCC and BA analysis highlights the poor agreement between the included strength tests. The recommendation to replace 1RM testing with isometric testing routines in the squat does not provide suitable concordance and is not recommended.

## Introduction

Maximum strength is considered a fundamental motor skill, influencing the specific performance in several sports. For example, in team sports such as soccer (Wisløff et al. 2004), handball (McGhie et al. 2020), and basketball (Warneke et al. 2022) high correlations between maximum strength and jumping or sprinting performance were reported. Furthermore, track and field (Loturco et al. 2018) and swimming (Wirth et al. 2022) athletes can improve their performance by exerting higher impulses against their respective resistance to optimize acceleration of their own body or an external resistance. Considering its crucial importance, many athletic training programs are designed to increase maximum strength, to augment sport-specific performance parameters, while diagnostics aim to screen the assumed success of the made effort (Chelly et al. 2009; Styles et al. 2016).

When aiming for maximize lower extremity speed strength, mostly to enhance jumping, sprinting or change of direction performance, the barbell squat is one of the most recommended exercises to implement in athletic training programs (Chelly et al. 2009; Lohmann et al. 2022; Styles et al. 2016). Indeed, available literature proofed strength increases between 10.0 and 32.3% dependent on used training periods, ranging between 6 and 12 weeks (Colquhoun et al. 2018; Green and Gabriel 2018; Lynch et al. 2020). However, especially in team sports, performance testing in a group setting is time consuming, and reaching maximal loads for 1RM testing is considered to induce fatigue. Therefore, the literature suggests to reduce the testing items to a very minimum and, based on correlation coefficients classified as high (> 0.7) between isometric and dynamic testing procedures, recommended the substitution of 1RM testing conditions by maximal isometric tests (McGuigan et al. 2006; McGuigan and Winchester 2008).

Indeed, the literature described isometric tests to be more time efficient and reliable while easy to standardize (McGuigan and Winchester 2008). Furthermore, due to less coordinative requirements compared to 1RM testing, they are considered safe and easy to conduct, thus, indicating substantial benefits in performance tests (Beckham et al. 2013; McGuigan et al. 2010). Consequently, McGuigan et al. (2010) proposed replacing dynamic testing of the squat and the bench press by using the isometric mid-thigh pull (IMTP), referring to high Pearson correlation coefficients of *r* = 0.69–0.97. However, when aiming to replace the dynamic testing by isometrics, two requirements need to be considered. First, a high agreement (low error) between testing procedures is necessary. Second, if there was a systematic error leading to limited agreement, it must be possible to calculate the maximal dynamic strength from the isometrics. To explore agreement, the magnitude of the unsystematic error can be performed by calculating the mean absolute error (MAE) (Willmott and Matsuura 2006) and the mean absolute percentage error (MAPE) (Hyndman and Koehler 2006), which can be supplemented by the concordance correlation coefficient (CCC) (Lin 1989) and graphically illustrated by a Bland–Altman (BA) analysis (Doğan 2018; Giavarina 2015). This procedure is also frequently recommended in methodological journals (Kim and Lee 2022; King and Chinchilli 2001) and already performed in different disciplines (Vetter and Schober 2018) to evaluate the agreement between different measurements (medical measurement devices) (Singh et al. 2020).

Due to high specificity in training-induced adaptations (Behm and Sale 1993) and an assumed disadvantageous transferability from isometric testing conditions to sports-specific performance parameters (Murphy and Wilson 1996), the usage of isometric tests could be questioned. Therefore, this study was conducted to first investigate the Pearson correlation coefficient and the ICC to ensure comparability to previous studies. Second, the corresponding MAE and MAPE were calculated and supplemented by the CCC and BA analysis to explore the replaceability of the 1RM testing by isometric condition, considering different knee joint angles in the squat (Hartmann et al. 2012) and the overall substitution of the 1RM squat testing by the IMTP.

## Materials and methods

To answer the research question, 53 healthy participants were recruited for participation, while 7 were excluded based on exclusion criteria. The maximum strength in the squat (isometrically and dynamically) as well as the IMTP were tested using a Smith machine. Tests were performed on two separated, non-consecutive days to avoid fatigue from testing. Dynamic tests were performed on the first day, isometric tests on the second testing day.

### Subjects

Considering a power of *β* *−* 1 error of 0.8, and an *α* error of 0.05, a priori G-Power sample size estimation suggested the inclusion of a minimum total sample size of *n* = 26 (Wagner et al. 2022). To enhance the statistical power, 46 healthy and recreationally trained participants (male: *n* = 33 age: 24.7 ± 3.6 years, height: 184.1 ± 6.2 cm, weight: 80.7 ± 7.7 kg, female: *n* = 13, age: 22.8 ± 2.2 years, height: 174.0 ± 6.2 cm, weight: 66.3 ± 9.5 kg) were recruited from the sport science program of the university campus. Participants were classified as recreationally trained if they performed at least three sessions of strength training per week or participated at least three times per week in the university sports program within the last year. All participants were considered experienced in resistance training by including squats in their regular training routine at least once per week within the last 6 months. Participants with a serious injury, leading to prolonged times of immobilization, within the last 6 months were excluded from the study. Seven participants were not able to perform a deep back squat or were not able to produce reliable isometric testing values and were, therefore, excluded from the data analysis.

### Procedures

#### Maximum strength testing

Before testing, a standardized general warm-up program was conducted. The participants were instructed to perform a 5-min ergometer cycling with 60 rpm, and 2 × 10 bodyweight squats. Under dynamic testing conditions, the participants used their individualized warm-up routine to reach 1RM in the squat, progressively increasing the weight. To prepare for isometrics, participants were instructed to perform submaximal isometric contractions against the fixed bar.

#### Isometric mid-thigh pull (IMTP)

To investigate the maximum strength in the IMTP, the barbell was fixed at the height of the patella of the participants, who were instructed to place themselves on a force plate (Kistler, Sindelfingen, Germany) with a shoulder-width stance. The participants were allowed to use a cross grip as the aim was not to investigate the grip strength as a limiting factor in the exercise. The back was to be held in a straight position, while the knees had to stay in line with the hips and ankle joints. The barbell was fixed in the Smith machine. The participant was instructed to perform a maximal pulling movement on the bar, pushing the scapula together and fixating the lower back in a straight position. The bar was positioned at the mid of the thigh to use the starting position of the second pull of the clean and jerk. For each trial of the IMTP, contractions were performed for 3 s with a rest of 2 min between each trial. The participants performed both movements until no maximum strength increase could be observed, with a minimum of three trials (Beckham et al. 2018; Kawamori et al. 2006).

#### Squat testing

**Fig. 1:**
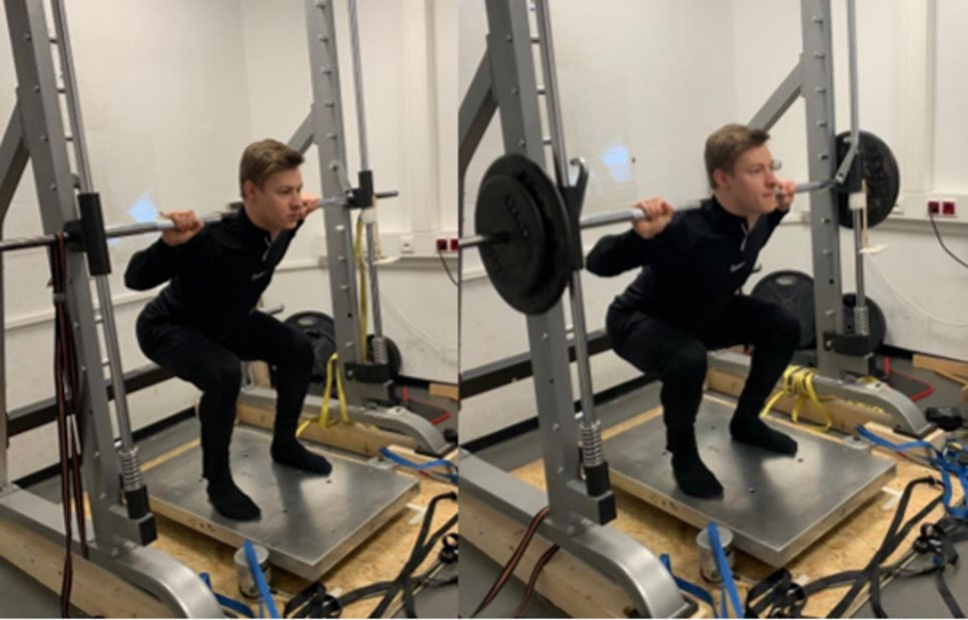
The 90° isometric squat test and the dynamic parallel 1RM squat test

Maximum squat strength was tested using the back squat movement. Therefore, the bar was placed on the trapezius muscle. For the isometric testing, the bar was fixed using the Smith machine after the participant took the corresponding position to perform the testing at the required knee joint angle (90°, parallel squat, deep squat). The maximum isometric strength was determined using a Kistler force plate (Kistler, Sindelfingen, Germany) (Fig. 1a).

For the 1RM testing, the participants started in an upright position, standing on the force plate. The testing was initiated by flexing the knees (Fig. 1b). Two experienced investigators rated the depth and movement execution independently and the trial was only considered appropriate if both investigators rated it positively. Between each trial, there was a rest of 2 min. The participants started with a weight equivalent to 70% of the isometric maximum strength value. Weight was added until no valid trial could be performed. The participant was allowed to try the maximum weight twice. A spotter ensured safety for the participants. For each trial of the squat contractions were performed for 3 s with a rest of 2 min between each trial. The participants performed both movements until no maximum strength increase could be observed, with a minimum of three trials.

### Statistical analysis

The data were analyzed using SPSS 28.0 (IBM, Ehningen, DE, Germany). The significance level for all statistical tests was set at *p* < 0.05. The descriptive statistics are presented as the mean (M) ± standard deviation (SD). Normal distribution was ensured using the Kolmogorov–Smirnov test. Reliability values were calculated providing ICC (95% confidence intervals (CI)) and the coefficient of variability (CV). To determine significant differences in the correlation coefficients between subgroups (male and female) the data were z-transformed according to the Fisher method. The difference between the two transformed values after standardization was assessed for significance (Ferreira and Zwinderman 2006). Results are provided after calculating bivariate two-tailed Pearson correlation analysis as well as presenting ICCs with 95% CIs between all maximum strength testing conditions, to ensure a comparability to previous study results. Moreover, the CCC (*ρ*_c_) (King and Chinchilli 2001; Singh et al. 2020) as well as BA analysis (Doğan 2018) are presented to adequately investigate the agreement between the different testing conditions. The MAE (Willmott and Matsuura 2006) is stated as a measuring of errors between paired observations evaluating the same parameter, while the MAPE (Hyndman and Koehler 2006) expresses accuracy, providing quantitative information about the deviation between two measuring procedures (Warneke et al. 2023). Therefore, both parameters can be stated to investigate the difference between a measured and predicted parameter and were commonly used to validate testing batteries (Gao et al. 2021). Since isometric measurement was performed on a force plate, while for 1RM calculation the added weight on the bar was used, the pure strength values (without inclusion of the bodyweight) were used for calculation.

The MAE and the MAPE were calculated using the following formula:

$$MAE=\frac{1}{n}*\sum_{i=1}^{n}\left|{x}_{i}-{y}_{i}\right|,$$

$$MAPE=\frac{100}{n}*\sum_{i=1}^{n}\left|\frac{{x}_{i}-{y}_{i}}{{x}_{i}}\right|.$$

with *x*_*i*_ being the values from the first testing, *y*_*i*_ being values from the second testing and *n* being number of the testing.

**Fig. 2:**
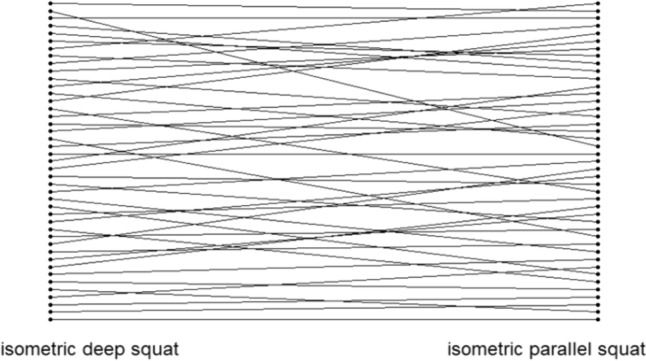
Exemplary illustrates Spearman’s ranking correlation coefficient, showing that the best participant in the one test is obviously not automatically the best participant in the other test

Concordance analysis was performed with “R” (Version R 64 4.1.3, Lucent Technologies, Dormagen, Germany). Lastly, as the agreement of two testing procedures could not be seen as given if in the one test, one athlete is ranked best, while in another testing procedure measuring the same motor ability, the same athlete is ranked worse, the Spearman rank correlation coefficient was determined and illustrated graphically (see Fig. 2).

## Results

Normal distribution of parameters was ensured using the Shapiro–Wilk test (*p* > 0.05). Since no significant difference between correlation coefficients between male and female participants was observed, the overall statistics including both sexes is provided in the following table. The descriptive statistics is provided in Table 1, and reliability is reported in Table 2.

**Table 1:** Descriptive statistics for included parameters

Parameter	Mean ± standard deviation (in *N*)
Isometric mid-thigh pull	1303.3 ± 333.5
Isometric deep squat	841.8 ± 285.4
Isometric parallel squat	777.7 ± 258.2
Isometric 90° squat	1124.4 ± 374.2
1RM deep squat	865.7 ± 244.7
1RM parallel squat	956.9 ± 262.8
1RM 90° squat	1212.9 ± 323.8

Isometric strength is provided without the body weight, 1RM maximal strength is provided as added weight to the bar * 9.81, to provide comparability in measurement units

*1RM* one repetition maximum

**Table 2:** Reliability of included testing procedures

Parameter	ICC [95% CI]	CV ± SD [95% CI] in %
Isometric mid-thigh pull	0.998 [0.997, 0.999]	1.29 ± 0.82 [1.05, 1.55]
Isometric deep squat	0.996 [0.993, 0.998]	1.90 ± 1.21 [1.54, 2.26]
Isometric parallel squat	0.966 [0.992, 0.998]	1.74 ± 1.07 [0.15, 0.21]
Isometric 90° squat	0.984 [0.971, 0.991]	2.02 ± 2.04 [1.52, 2.66]
1RM deep squat	0.996 [0.992 0.998]	1.76 ± 2.05 [1.18, 2.41]
1RM parallel squat	0.997 [0.995, 0.998]	0.97 ± 1.74 [0.49, 1.48]
1RM 90° squat	0.995 [0.991, 0.997]	1.05 ± 2.01 [0.49, 1.70]

*1RM* one repetition maximum, *ICC* intraclass correlation coefficient, *CV* coefficient of variability, *SD* standard deviation, *95% CI* 95% confidence interval

Table 3 provides the statistics including Pearson correlation coefficients (*r*), ICCs, CCCs, MAPEs, MAEs, and the maximal percentage error (MPE) between the parameters, indicating moderate to high magnitude correlations with *r* = 0.645–0.961, ICC = 0.645–0.86. Correlation coefficients between IMTP and isometric squats range from 0.688 to 0.833 and between IMTP and 1RM squat testing from *r* = 0.701–0.743 showing a moderate to high relationship between these testing conditions. There were moderate to high correlation coefficients between isometric squat test and dynamic testing conditions ranging from *r* = 0.645–0.961. The highest correlation coefficient could be determined between 1RM testing in the deep squat and parallel squat with *r* = 0.961.

**Table 3:** Measurements of agreement between isometric and dynamic testing conditions

Parameters	*r*_*p*_	ICC (95% CI)	*r*_*s*_	CCC *ρ*_*c*_ (95% CI)	MAE (N)	MAPE (%)	MPE (%)
IMTP/1RM deep squat	0.722*	0.688 (0.492–0.818)	0.736 (0.552–0.851)	0.32 (0.19–0.44)	444.17	57.71	74.47
IMTP/1RM parallel squat	0.715*	0.695 (0.502–0.823)	0.696 (0.493–0.827)	0.41 (0.25–0.55)	363.47	27.19	51.76
IMTP/1 RM 90° squat	0.691*	0.691 (0.496–0.820)	0.683 (0.475–0.819)	0.67 (0.47–0.8)	219–11	16.60	46.88
Isometric deep squat/1RM deep squat	0.638*	0.630 (0.410 –0.781)	0.664 (0.447–0.807)	0.63 (0.41–0.78)	175.75	23.77	125.90
Isometric parallel squat/1RM parallel squat	0.828*	0.828 (0.704–0.903)	0.793 (0.641–0.885)	0.67 (0.51–0.78)	189.27	29.86	125.90
Isometric 90° squat/90° 1RM squat	0.723*	0.716 (0.532–0.835)	0.687 (0.480–0.822)	0.69 (0.51–0.82)	218.63	22.44	74.25

*1RM* one repetition maximum, *r*_*p*_ Person correlation coefficient, *r*_*s*_ Spearman ranking correlation coefficient, *ICC* intraclass correlation coefficient, *CCC ρ*_*c*_ concordance correlation coefficient, *MAE* mean absolute error, *MAPE* mean absolute percentage error, *MPE* maximal percentage error, * = *p* < 0.001

**Fig. 3:**
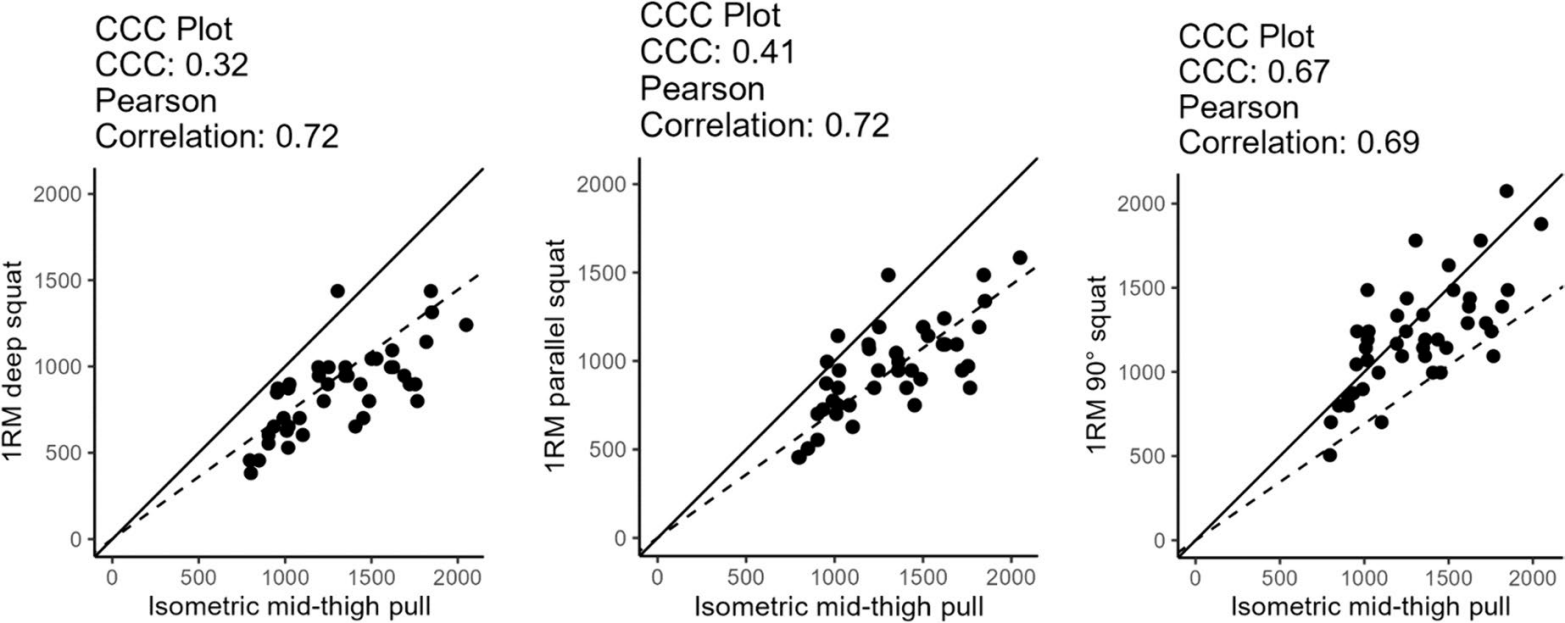
Graphical illustration of the concordance correlation coefficient by Lin (1989) for the isometric and dynamic squat test and the IMTP and the 1RM deep squat

**Fig. 4:**
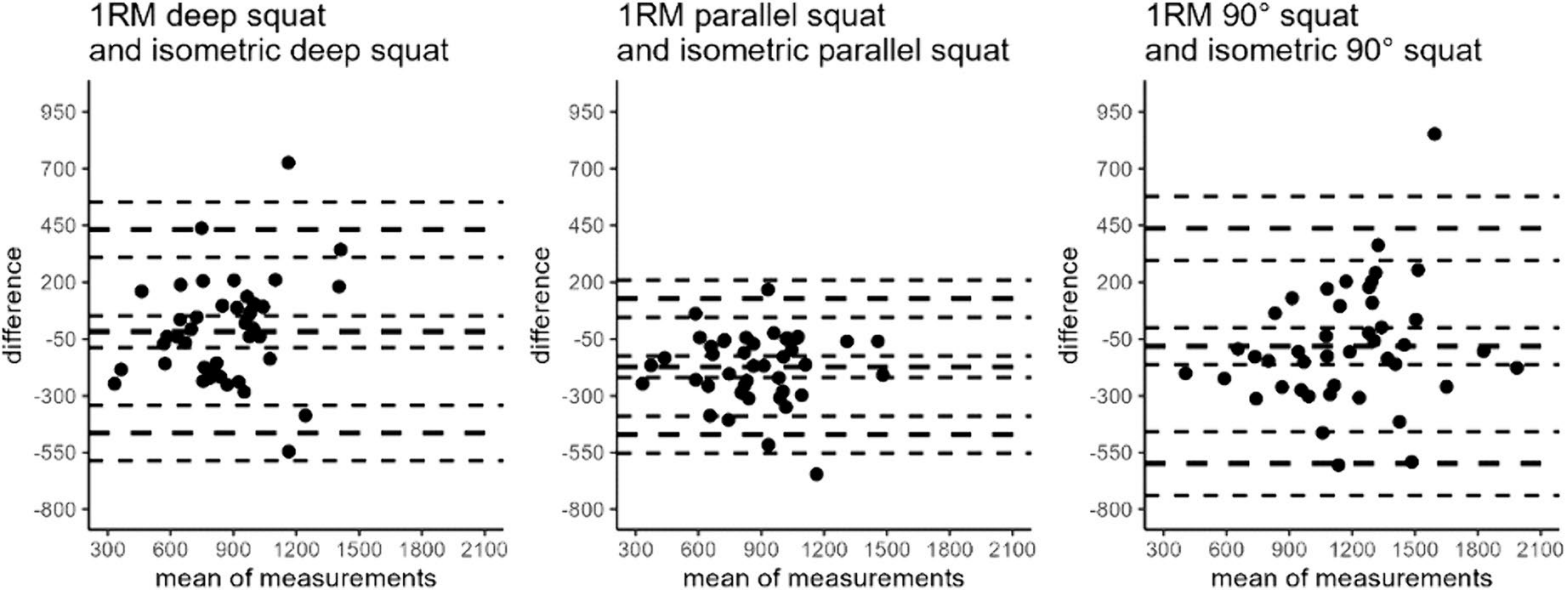
The agreement between measurements for **a** 1RM deep squat and isometric deep squat, **b** 1RM parallel squat and isometric parallel squat, **c** 1RM 90° squat and **d** isometric 90° squat and the IMTP and the 1RM deep squat

The performed agreement analysis including the MAE and MAPE are provided in Table 3, while graphically illustrated in Fig. 3 showing the CCC as well as Fig. 4 the BA analysis. Furthermore, Fig. 4 exemplary provides a graphical presentation of the Spearman ranking correlation coefficient (*r*_*s*_).

In the current study, the results showed correlation coefficients ranging from *r* = 0.638–0.828 with ICCs of 0.630–0.828 between isometric and dynamic maximum strength tests in the squats. The corresponding agreement analysis showed a MAE of 175.75–218.63 N with a MAPE of 22.44–29.86%. The correlation coefficients between the IMTP and the 1RM squat ranged from *r* = 691–0.722 with ICCs of 0.683–0.695, with MAE of 219.11–444.17 N and MAPE between 16.60 and 57.71%. The graphical illustration of the Spearman correlation coefficients is included to demonstrate the level of heterogeneity considering *r*_*s*_ = 0.664–0.793.

## Discussion

In the literature, the replacement of 1RM squat tests by isometric tests (such as the IMTP) was suggested based on correlation coefficients with *r* > 0.7. Regarding the replaceability of the 1RM squat test using the IMTP, McGuigan et al. (2010) stated “Strength and conditioning coaches and other practitioners with access to a force plate can consider using the isometric mid-thigh pull test as a potential alternative to traditional 1RM testing. In recreationally trained subjects, it appears to correlate extremely well with both the 1RM squat and bench press”.

In the current study, the results showed correlation coefficients ranging from *r* = 0.638–0.828 with ICCs = 0.630–0.828 between isometric and dynamic maximum strength tests in the squats, which is in accordance with previous literature (Bazyler et al. 2015). Considering *ρ*_c_ = 0.63–0.69 substituted by the graphical illustrations of the CCC and BA analyses (Figs. 2, 3, 4, 5) a high agreement between isometric and dynamic strength testing in the squat seems questionable, especially taking into account the MAEs ranging from 175.75 to 218.63 N with corresponding MAPEs between 22.44 and 29.86%. In accordance with previous research, correlation coefficients between the IMTP and the 1RM squat were classified moderate to high *r* = 0.691–0.722 with ICCs of 0.688–0.695 (Spiteri et al. 2014; Townsend et al. 2019), however, showing MAEs of 219.11–444.17 N and MAPEs of 16.60–57.71%. Claiming a substitution of the 1RM squat test by the IMTP (McGuigan et al. 2006, 2010; McGuigan and Winchester 2008), the reported measurement error arising from limited agreement must be considered and reviewed from a practitioner’s point of view.

When classifying correlation coefficients in sport and exercise science, authors commonly refer to Cohen (1988). Interestingly, it seems that authors neglect the recommendation to interpretate correlations in the respective topic. Examples provided by Cohen (1988), mostly refer to behavior sciences and psychology when describing a relationship between two measured values. When aiming to explore the replaceability of two measurements, we are not interested in the relationship, but the agreement of the measurements of interest, as correlations and agreement are two very different constructs (Liu et al. 2016). Accordingly, a recent review using simulated data provided measurement errors corresponding to different ICCs, applicable to strength testing conditions (Warneke et al. 2023), challenging the classification of correlation coefficients and ICCs > 0.7 as high. In accordance with the presented results, under scientific criteria, assuming an average maximal strength increase of up to 32.3% using common intervention periods of 6–12 weeks, a measurement error between dynamic and isometric conditions of 22.4–29.9% in the squatting test seems intolerably high.

Similar approaches are provided by Cataldi et al. (2022) who suggested to rate CCC values *ρ*_*c*_ < 0.9 as poor agreement in the field of body composition measurements, while a moderate agreement would be given with *ρ*_*c*_ = 0.90–0.95, a substantial agreement with *ρ*_*c*_ = 0.95–0.99, with an almost perfect agreement with *ρ*_*c*_ = 0.99. Accordingly, Dominguez Jimenéz (2016) described *ρ*_*c*_ = 0.68–0.77 as “no agreement”. With a *ρ*_*c*_ = 0.70, a MAPE of at least 17.36% and a MAE of 304 N can be assumed (Warneke et al. 2023), this claim can be supported also for strength testing. Based on listed classifications, the findings of the current concordance analyses could be classified as no—poor agreement between isometric and dynamic testing conditions. Accordingly, even with high correlation coefficients of > 0.7, considering the comparably high MAE and MAPE values of up to 57.71%, it seems questionable to classify Pearson correlation coefficients as high when aiming the replaceability of two measurements.

The practical relevance of the topic is underlined by a recent systematic review with meta-analysis performed by James et al. (2023) showing significant differences between isometric and dynamic strength increases in response to resistance training. Accordingly, the authors suggested both strength qualities to represent different strength constructs, consequently, in accordance with the presented results, separated testing protocols are required. Differences between isometric and dynamic testing conditions can possibly attributed to different factors. Assuming angle specificity as a moderating factor for isometric strength (Bazyler et al. 2015) would be an appropriate explanation of the comparatively low concordance between different angles in isometric testing and full ROM 1RM testing conditions (Wagner et al. 2022). Furthermore, angle specific training adaptations can be assumed, which must be considered in testing protocols. For example, Yahata et al. (2021) stretched the calf muscles exclusively reporting strength increases in the stretched ankle angle, while the neutral as well as the plantar flexed strength assessment did not show significant increases. Accordingly, the presented results show the highest agreement in similar knee angles (IMTP and 90° isometric squat with a MAE: 219.11 N, MAPE: 16.60%) and movement pattern (90° isometric squat and 90° 1RM squat MAE: 218.63 N, MAPE: 22.44%), while the worst agreement was found for the IMTP and the 1RM deep squat (MAE: 444.17 N, MAPE: 57.71%).

A further considerable aspect when performing diagnostics which could cause intolerable measurement errors is the contraction type. There seems to be a difference between isometric and dynamic testing conditions, possibly depending on familiarization with testing conditions (Drake et al. 2019). Noticeably, McGuigan et al. (2006) reported very high correlations between isometric testing and 1RM squat and bench press tests with elite wrestlers, who can be assumed to be familiar with isometric contractions while in a general population (not specialized on isometric muscle contractions) lower correlations were observed (Drake et al. 2019). A high specificity in testing conditions was earlier highlighted by Behm and Sale (1993) pointing out a high contraction velocity specificity. These studies suggested a high relevance of familiarization with testing conditions and support the demand for designing highly specific tests (Warneke et al. 2023). Although it seems that both contraction types require similar activational patterns, apart from the synchronically maximal fiber recruitment, maximal frequent (up to) tetanic activation depends on the optimum preset of the muscle spindle, which is influenced by the alpha-gamma-co-activation in regard of increased fusi-motor drive (Hagbarth et al. 1986; Holtermann et al. 2007). This preset of the central neural drive depends on the expected contraction form (Grabiner and Owings 2002) and the load-height that is meant to be accelerated (Hernández-Davó et al. 2015). Participants in studies Grabiner and Owings (2002) had to perform an isometric contraction in the isokinetic dynamometer, which was followed by a maximal eccentric OR maximal concentric contraction. The eccentric contraction led to significant lower initial EMG-amplitude (100 ms before execution, which means in the isometric condition) compared to the concentric one.

When the following contraction form was insufficiently announced, subjects showed the initial activation pattern in the isometric phase, which corresponded the one which was expected. Accordingly, the expectation of performing a following movement seems to determine central nervous activation patterns, making it complicated to sufficiently active the muscles if the following contraction form was unfamiliar. As a consequence, it could be hypothesized that subjects which are not familiarized with isometric contractions have difficulties in performing maximal effort against an immovable weight. Therefore, investigators and coaches should be aware that testing isometric maximum strength for reasons of time efficiency, does not allow a (precise) statement about dynamic force development in more sport-specific movements. Since in most sports (except for a low number of specific sports) athletes perform mostly dynamic movements and are not accustomed to isometric muscle contractions, dynamic testing conditions seem more appropriate, especially when aiming to screen the results of a dynamic squat training intervention.

The described statistics indicates that there is only poor agreement between IMTP and 1RM squatting conditions as well as between isometric and dynamic testing conditions in the squat. In addition, the Spearman ranking correlation coefficient was calculated and exemplary illustrated in Fig. 4. Reviewing the graphic underlines the limited possibility to predict the 1RM maximum strength from the isometric testing, since there is no consistency in the ranks (rank six in the isometric test is not necessarily the rank six in the dynamic testing). Therefore, there is no accurate and precise determination of the exact strength value from isometric to dynamic testing.

### Limitations

Based on exclusion criteria, it was not possible to include a well-balanced distribution between males and females in the current study. However, since no significant difference in the correlations was observed, no sex specificity was initially assumed. Furthermore, the inclusion and exclusion criteria allowed the participation of subjects from different fields of sports with a potential influence on the correlations, especially since an influence of familiarization is hypothesized. Using more homogenous participants could lead to different results; however, it can be assumed that a more heterogenous field of testing subjects would even overestimate the correlation coefficients. Another limitation can be seen in the determination of the 1RM strength values, since those were calculated by using the kg value multiplicated with 9.81, to reach the same unit as used in the isometric testing. Otherwise, the calculation of the ICC, CCC and BA would obviously produce problems, just because of using different units. In the presented study, only the agreement between two different testing conditions was examined. However, no statement about the possibility to calculate, for example, the 1RM squat strength using the corresponding maximum isometric strength value. However, because of the comparative high variability, which can be reviewed in the Bland–Altman Plots, there are reasonable doubts about an accurate calculation of 1RM values based on isometric strength values in the squat.

#### Practical application

Considering the comparatively low expected increases in strength capacity in response to short intervention periods of a few weeks, an “almost perfect agreement” (Domínguez Jiménez and Fernández Suárez 2016) seems necessary between two measurement devices to replace one of those with the other. There are no general recommendations available about the tolerable measurement error, however, even with a CCC of *ρ*_*c*_ = 0.96, a MAPE of 11.11% with a MAE of 91.26 N was found in the current study. Under scientific criteria, assuming a strength increase of up to 32.3% (Green and Gabriel 2018; Lynch et al. 2020), a MAPE of approximately one-third seems intolerably high. Therefore, aiming to replace 1RM testing conditions, high magnitude agreement between measurement devices and higher expected strength increases are required. This can be reached, for example, with longer training interventions, since Sander et al. (2013) showed strength increases in 1RM back squat of about 101–291% (*d* = 5.5–5.6) with a 2-year strength training intervention, while Keiner et al. (2022) enhanced maximum strength with 12% (*d* = 1.0) by performing a 10 month strength training intervention in soccer players.

## Conclusion

The demand to replace 1RM testing conditions with isometric tests in the squat or the replacement of the 1RM squat test by the IMTP seems without adequate evidence. Previous literature stating a high agreement between isometric and dynamic testing conditions using Pearson correlations or ICCs (> 0.70) should be questioned, since the classification of the correlation coefficients should be performed based on the agreement or measurement error between the testing conditions. The presented study, however, showed intolerable high MAE and MAPE values even with high correlation coefficients, supporting the initial demand for a content dependent classification of the correlation coefficient, if the aim is to investigate the agreement. Therefore, further agreement calculations should include a quantification of the error between the two testing conditions to improve the classification of the correlation analysis, targeting the agreement of two methods.

## Data Availability

Original data can be provided due to reasonable request.
